# Non-isothermal drying kinetics of human feces

**DOI:** 10.1080/07373937.2019.1670205

**Published:** 2019-10-01

**Authors:** T. Somorin, B. Fidalgo, S. Hassan, A. Sowale, A. Kolios, A. Parker, L. Williams, M. Collins, E. J. McAdam, S. Tyrrel

**Affiliations:** a Department of Chemical & Process Engineering, University of Strathclyde, Glasgow, UK;; b School of Water, Energy and Environment, Cranfield University, Cranfield, UK;; c Naval Architecture, Ocean & Marine Engineering, University of Strathclyde, Glasgow, UK

**Keywords:** Drying, kinetics, human feces, Nano Membrane Toilet, thermal analysis

## Abstract

The non-isothermal drying behavior and kinetics of human feces (HF) were investigated by means of thermogravimetric analysis to provide data for designing a drying unit operation. The effect of heating rate and blending with woody biomass were also evaluated on drying pattern and kinetics. At low heating rate (1 K/min), there is effective transport of moisture, but a higher heating rate would be necessary at low moisture levels to reduce drying time. Blending with wood biomass improves drying characteristics of HF. The results presented in this study are relevant for designing non-sewered sanitary systems with in-situ thermal treatment.

## 1. Introduction

According to the Joint Monitoring Programme for Water Supply, Sanitation and Hygiene,^[^^1^^]^ 32% of the population had no access to basic sanitation services in 2015 and achievement of universal basic sanitation by 2030 is a major challenge in many developing countries. In these countries, more than 90% of the feces generated are disposed into surrounding waters without treatment with health and environmental risks.^[^^2^^]^ The situation is less critical in developed countries; nevertheless, current sewered sanitary facilities present huge costs, high energy requirements, and environmental implications. Various initiatives are being developed to establish next-generation sanitary systems that can ensure safe and affordable treatment without compromising on sustainable use of resources and environment.^[^^3a^^]^ Non-sewered systems without external water and energy supply are emerging as one of the solutions to poor sanitation.

Human feces (HF) is a biomass which consists of a mixture of undigested fat, protein, water, polysaccharide, bacterial biomass, gut secretions, cell shedding, and ash.^[^^4^^]^ Feces can be converted into energy via thermochemical conversion processes such as gasification and combustion. Some novel concepts of sanitary systems include in-situ thermal treatment of the feces with a two-fold objective: to treat and eliminate the HF waste, and to produce energy for running a self-sufficient unit. An example of this approach is the Nano Membrane Toilet (NMT) which is being developed at Cranfield University as a response to the “Reinvent the Toilet Challenge” set by Bill & Melinda Gates Foundation.^[^^5^^,^^6^^]^ The NMT includes a dryer to reduce the moisture content of HF and a micro-combustor to convert the residual solids into useful energy. The design of both stages is driven by the achievement of a complete conversion of HF into heat and flue gas as the end products and the efficiency of the process.

As part of ensuring consistent and reliable operation, and like other fuels, the HF feed must be conditioned prior to thermochemical conversion. One of the key parameters that need to be considered is the moisture content. The use of a wet fuel may cause delay or failure of ignition since part (or all) of the heat in the system is consumed by water vaporization. The moisture content in HF is typically between 70 and 82 wt.%,^[^^3b^^]^ which indicates that drying is required prior to thermal conversion processes.^[^^7^^]^ The process of drying involves the removal of water content from a material ensuring the viability of the dried material for subsequent processing. The drying process typically involves three stages: (a) the warm-up period where the material is heated up to the wet bulb temperature and the moisture evaporation rate from the surface of the material is higher than the diffusion rate of inner moisture of the sample, (b) the constant rate period where the temperature remains constant and the diffusion of inner moisture is equal to the surface moisture evaporation rate, and (c) the falling rate period in which the drying rate decreases as the inner moisture migrates and moisture content in the surface decreases. These stages are defined by physical states of water: free water, interstitial water, surface water, and intracellular water, and affected by the type and strength of the chemical bonds in water molecules. Only the free water and a part of the interstitial water are removed by conventional dewatering processes.^[^^8^^]^ Establishing the drying stages of fecal solids is thus important for predicting drying profiles.

Thermogravimetric analysis (TGA) has been commonly used to study the drying of biomass.^[^^9–12^^]^ To exemplify this, Qian et al.^[^^13^^]^ investigated isothermal drying characteristics of sewage sludge using a thermogravimetric analyzer with no gas injection to obtain drying characteristics in the condition similar to that of the conductive indirect-heating dryer with agitation. Chen et al.^[^^14^^]^ examined the drying performance and the effect of compositional changes of foam pretreated sewage sludge to establish the optimum operating pretreatment conditions. Zhang and Chen^[^^15^^]^ applied isothermal and non-isothermal methods to establish the dominant mechanism for drying municipal sewage sludge. These studies have employed one or more of the following: used Arrhenius-type equations to establish the temperature dependency of moisture diffusivity; explored different material compositions, reaction atmospheres, temperatures, and heating rates to establish the optimum drying performance; and determined the activation energy and frequency factor for the various stages of drying. They have proven that TGA can enhance the understanding of drying performance and kinetics, but more information on the drying behavior of HF is required to achieve efficient designs of sanitary systems with in-situ thermal treatment. Current studies on drying of HF are limited to physical processes^[^^16^^,^^17^^]^ with limited or no information on drying kinetics. And while sewage sludge materials may to some extent contain HF, they are processed solids and by-products of advanced wastewater treatment processes. They contain a wide variety of materials from domestic and commercial facilities including chemicals and metal leachates.^[^^18^^,^^19^^]^ They differ in material composition, even, micronutrients and heavy metals are higher in quantity and diversity. More information is thus required to assess potential differences in the drying behavior of HF^[^^20^^]^ and ascertain their drying kinetics.

The aim of this work is to investigate the non-isothermal drying kinetics of HF by means of thermal analysis. The influence of drying on HF was studied by testing three sample types. In addition, the effects of heating rate and material blending of HF and woody biomass (WB) were evaluated. The research provides data for understanding the drying kinetics of HF as an initial stage during thermochemical conversion processes, which is fundamental to appropriately design an onsite, non-sewered sanitary systems.

## 2. Materials and methods

Fresh HF was collected and stored in a freezer at 188 K (−85 °C) to prevent microbial degradation. Before testing, the frozen samples were thawed at room temperature and mixed until a uniform consistency was obtained. Three different types of HF as classified in the Bristol Stool Chart (BSC) were analyzed: BSC2, BSC4, and BSC5. The BSC2 is described as a “lumpy, hard stool”, while the BSC4 and BSC5 are referred to as “smooth, soft, easily passed feces with or without clear cut edges”.^[^^20^^]^ Blends of HF with WB samples with 20, 40, 60, and 80 wt.% of HF were also analyzed. Drying behavior was evaluated in a Perkin Elmer “Pyris 1” thermogravimetric analyzer. The non-isothermal tests were performed on 20 ± 0.5 mg of the sample at airflow rate of 60 mL/min. The temperature was first set at 303 K for 2 min for weight stabilization, and then constantly heated at a rate of 10 K/min up to a temperature of 423 K. The sample was kept at the 423 K for 8 min to ensure the complete removal of water from the sample. Similar sets of experiments were carried out at heating rates of 1 K/min and 5 K/min. At smaller quantities, there were variations in test results due to the high moisture content of the samples, thus sample weight was pre-selected to ensure test repeatability.

The moisture content in wt.% for each sample was calculated using Equation (1).

(1) $$m(\text{wt}.\%,\text{arb})=\frac{m_{0}}{m_{s0}}\cdot 100$$

where *m* is the moisture content in as-received basis, *m*_0_ is the initial moisture content, *m_s_*_0_ is the initial mass including the moisture content.

The moisture ratio (MR) of the samples was calculated as given by Equation (2).

(2) $$\text{MR}=\frac{m_{t}-m_{f}}{m_{0}-m_{f}}$$

where *m_t_* is the time-dependent moisture content, and *m_f_* is the final moisture content (considered zero in this work). Samples are labeled as BSCX–Y, where X refers to the type of HF (i.e. X = 2, 4, or 5), Y refers to the ratio of HF in the analyzed sample (i.e. Y = 20, 40, 60, and 80), where samples are mixed. Table 1 summarizes the moisture content of the samples at different conditions.

**Table 1.** Moisture content of the samples as determined by TGA at 10 K/min.

Sample ID	Amount of HF (wt.%)	*m* (wt.%, arb)
BSC2-20	20	14.5
BSC2-40	40	15.9
BSC2-60	60	21.1
BSC2-80	80	25.2
BSC2	100	53.8
BSC4-20	20	8.6
BSC4-40	40	20.6
BSC4-60	60	31.8
BSC4-80	80	43.6
BSC4	100	56.9
BSC5-20	20	9.8
BSC5-40	40	18.4
BSC5-60	60	33.2
BSC5-80	80	44.9
BSC5	100	62.4
WB	0	8.9

### 2.1. Determination of kinetics

Non-isothermal kinetics were investigated through model-free and model-fitting methods. The Ozawa-Flynn-Wall (OFW) approach was selected as the model-free method^[^^11^^,^^12^^,^^15^^]^—Equation (3). These methods allow investigating the drying kinetics and activation energy without determining the reaction order.

(3) $$\;\text{log}\;\beta =\;\text{log}\;\frac{AE_{a}}{\text{Rg}\left(\alpha \right)}-2.315-0.457\frac{E_{a}}{\text{RT}}$$

(4) $$\alpha =\frac{m_{s0}-m_{\text{st}}}{{m_{s0}-m}_{\mathrm{sd}}}$$

where *α* is the degree of conversion at any time calculated by Equation (4), *g*(*α*) is the integral representation of the drying reaction model, *β* is the heating rate in K/min, *E_a_* is the activation energy in kJ/mol, *A* is the pre-exponential factor, and *m_st_* is the time-dependent mass of the sample including moisture content. A reference value of the activation energy can be determined for each period of the drying process by plotting log *β* vs. 1/*T* at a certain conversion degree. The model-fitting methods allow determining the drying reaction order that cannot be established by means of the model-free models. In this work, the method based on Coats-Redfem (CR) equation was used. CR is a commonly applied model-fitting method that requires the assumption of the integral drying model *g*(*α*) to be solved—Equation (5).

(5) $$\;\text{ln}\;\frac{\text{g(α)}}{T^{2}}=\;\text{ln}\;\frac{\text{AR}}{\beta E_{a}}-\frac{E_{a}}{\text{RT}}$$

A number of *g*(*α*) expressions has been defined as common mechanisms functions for non-isothermal solid reactions, which are applicable to drying model.^[^^15^^,^^21^^]^ In this work, *g*(*α*) in Equation (5) was substituted with the common functions. The resulting values for ln[*g*(*α*)/*T*^2^] were plotted vs. 1/*T* to obtain *E_a_* and *A* values of the samples in each period of the drying. The preferred drying model was selected based on the comparison of the *E_a_* values with the reference value obtained from OFW equation.

## 3. Results and discussion

### 3.1. Effect of fecal types and heating rates

**Figure 1.**
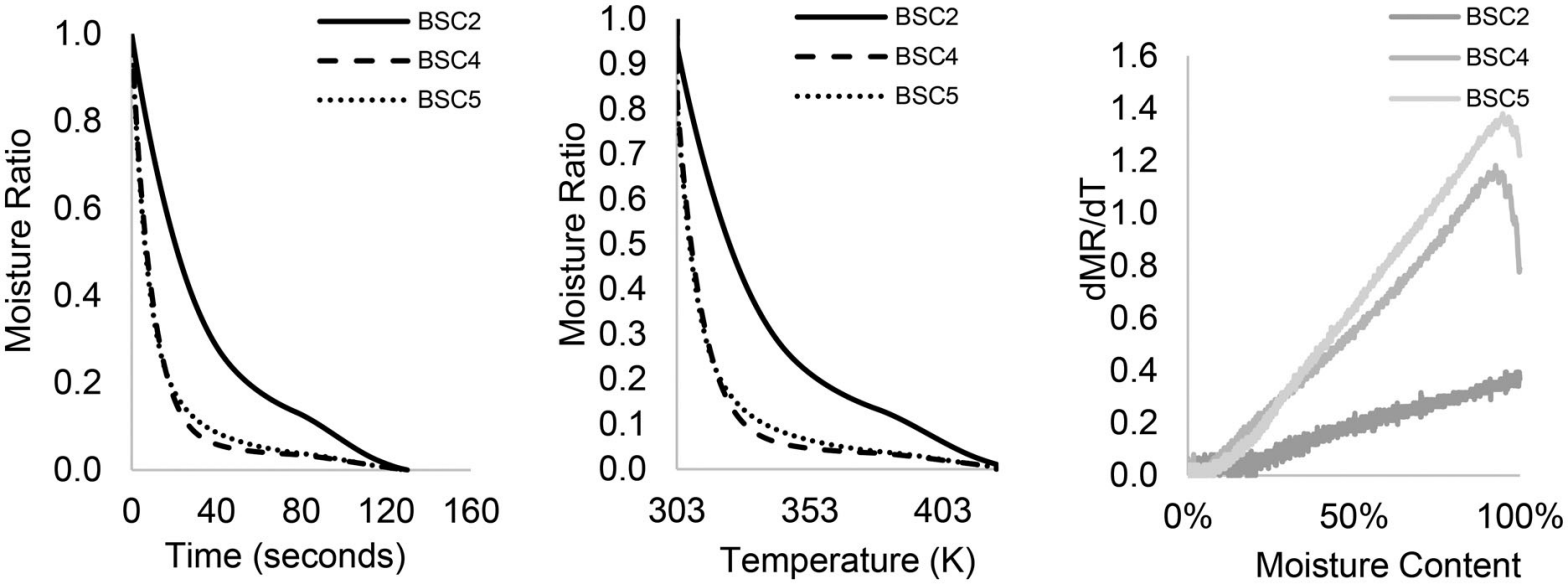
The non-isothermal drying curves of HF (BSC2, BSC4, and BSC5) at 1 K/min: (a) moisture ratio vs. time, (b) moisture ratio vs. temperature, and (c) drying rate vs. moisture content.

**Figure 2.**
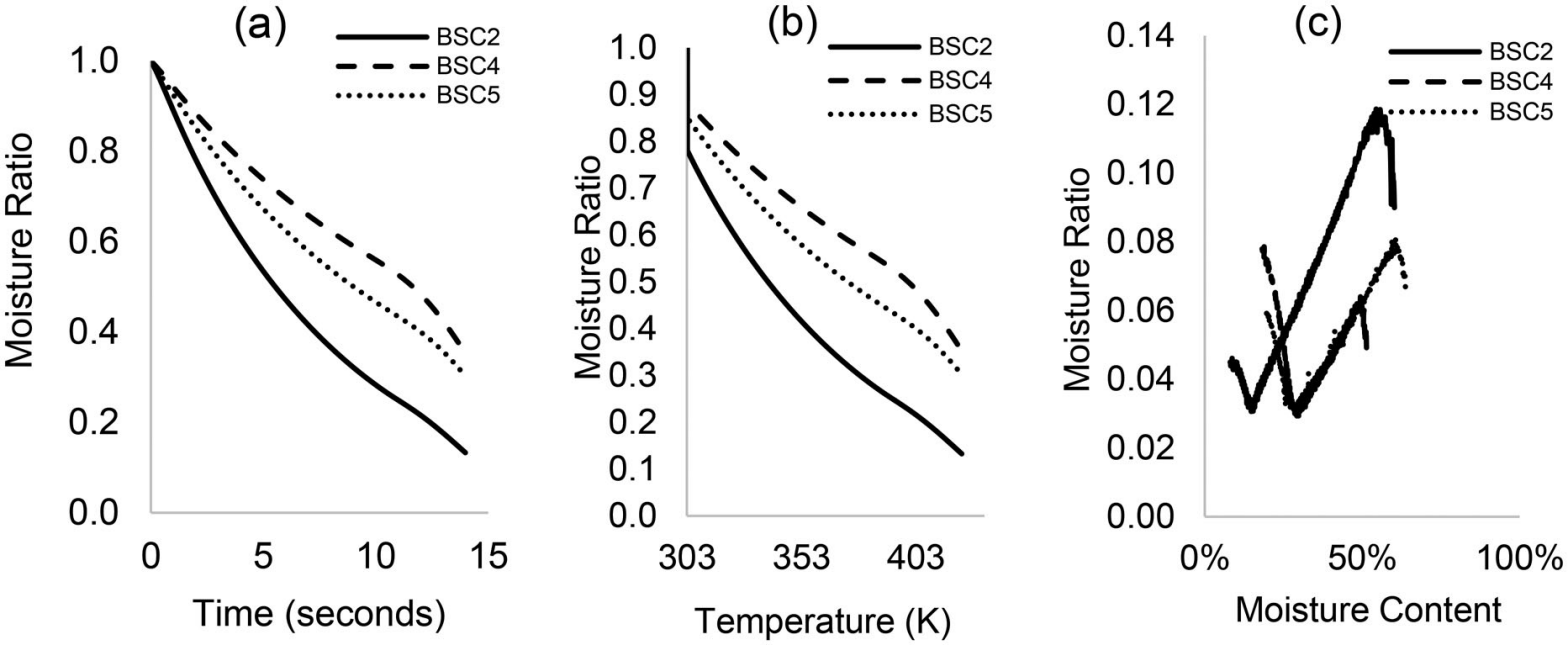
The non-isothermal drying curves of HF (BSC2, BSC4, and BSC5) at 10 K/min: (a) moisture ratio vs. time, (b) moisture ratio vs. temperature, and (c) drying rate vs. moisture content.

The non-isothermal drying behavior of the fecal types BSC2, 4, and 5 and at 1 and 10 K/min are illustrated in Figures 1 and 2 by means of (a) MR vs. time, (b) MR vs. temperature, and (c) drying rate (MR derivative) vs. moisture content.

Figure 1(a,b) show that the rate of moisture loss is similar for BSC4 and 5 samples, but different to those presented for BSC2. For all HF samples and at 1 K/min, the drying curve decreased continuously up to ∼350 K for BSC2 and ∼345 K for BSC4 and BSC5, which is followed by a constant rate period. At 323 K (Figure 1(a)), about 80 wt.% of the moisture in BSC4 and 5 had been given off, but for the same temperature, only 50 wt.% of the moisture was lost in BSC2. Considering the above-mentioned drying stages (warm-up, constant, and falling rate period), all the sample types exhibited similar drying curves. None of the curves had an initial constant rate period, but the removal of moisture from the solid matrices showed a significant falling rate tendency. These observations are confirmed in Figure 1(b) with a linear decrease in drying rate for all HF types until moisture was completely removed. The BSC2 samples had the least drying rate, which was at most 0.34 mg/min. For all the samples, the falling rate period occurred in two stages with no intermittent constant rate period, but there was a prior lagging “warm-up” period in BSC4 and 5 samples, which was absent in BSC2—Figure 1(b).

The observed differences in the drying profiles among the three samples can be attributed to the varying levels of compactness of the solids. These results suggest that the moisture profiles for these samples are different, perhaps the process by which moisture is being removed from the interior and the surface occurs via different phenomenon. And though, the differences in the moisture content between BSC2 and 4 are not as much as those between BSC4 and 5, the degree of compactness in the BSC2 sample has implications on moisture transport and diffusion processes and can impede the migration of water from the inside to the surface of the sample. Seader and Henley^[^^22^^]^ have described the phenomenon behind the falling rate period for different types of solids. Unlike granular materials, e.g. sand, where moisture is held freely in open pores, moisture is often trapped, locked or bound to chemical compounds in organic materials. Under heating conditions, material properties change, leading to shrinking, hardening, cracking during drying and swelling when exposed to moisture. In these cases, drying causes moisture to be released and the drying rate decreases linearly with time, as also observed in this study.

The lagging period observed in some samples characterizes an initial heating segment when the sample is being heated to a wet-bulb temperature and moisture loss is at its minimum. The absence of a constant rate period prior and between falling rate regimes suggest that there is a limited layer of free water on the surface of the material that can be removed by dewatering. It shows that the initial moisture content of the samples is below the “critical moisture content”.^[^^23^^,^^24^^]^ It also implies that there is a thin boundary layer for drying that favors evaporation from within the material rather than from the surface.^[^^25^^]^ Here, the migration of water to the surface is typically achieved by capillary action and by progressive evaporation-condensation, processes that are accompanied by a temperature gradient within the material. The multiple falling rates with no intermittent constant rate characterize the drying of HF as a partially wet and dry process. In the first falling rate, the proportion of the wetted area reduces significantly with time and as the moisture content of the sample decreases while moisture is progressively moved to the surface. In the subsequent falling rate, the surface of the material is free of water, evaporation front recedes and divides the material into a wet and dry region, but the material still contains other water forms.^[^^26^^]^ The slow, progressive movement of water and evaporation within the material, then causes drying rate to reduce and induces shrinking, hardening and cracking, as surface temperature increases. In this study, the slow drying rate causes drying time to increase significantly, almost five-fold (Figure 1(a)).

At heating rate (10 K/min)—Figure 2(a), similar drying curves are observed with MR reducing as temperature increases; however, with reverse effect on fecal types. At 323 K, MR was higher than 70 wt.% in BSC4 and 5 and up to 60 wt.% in BSC2, which implies that a higher heating rate has not favored drying. The drying rate curves in Figure 2(b) exhibited a falling rate tendency up to temperatures of about 385 K and all the samples had an initial warm-up period. The first falling rate period is characterized by a linear decrease in the drying rate with increasing time and moisture levels, corresponding to temperatures between ∼303 and 385 K. However, in the second falling rate period (>385 K), the drying rate increased with time and with moisture levels, which is dissimilar to the constant drying rate observed at 1 K/min.

The overall reduction in drying rate at relatively high heating rates can be attributed to mass and heat transfer limitations. Here, there is less gradual and effective heat transfer into the inner part of the sample, which impedes the release of moisture during drying.^[^^27^^]^ The linear decrease in drying rate with time as observed in the first falling rate period and subsequent increase in drying rate for all the samples suggest that there is a lower critical moisture content in the samples, which varied for all sample types: 25 wt.% (BSC2), 45 wt.% (BSC5), and 58 wt.% (BSC4). This critical moisture content reflects the time it takes for the moisture to travel from the interior to the receded evaporation front and the magnitude of resistance in heat and mass transfer for the various moisture forms. Here, surface water is completely removed, and the rate of evaporation is greater than the rate of diffusion. These observations are important to properly design a drying unit operation dealing with HF as its performance may change considerably depending on the type of HF. The minimum drying temperature at 10 K/min is ∼380 K (BSC2), and ∼390 K in the case of BSC4 and BSC5. The increase in drying rate at high temperatures as observed at 10 K/min may be related to rapid heating that causes a temperature gradient in the sample and gradual release of moisture. More so at higher temperatures, transport mechanisms are overtaken by thermal events, which improve drying rates, but complete drying is not achieved within the non-isothermal heating regime as the case of 1 K/min. The reduced moisture removal for BSC4 and 5 suggest limitations to the effective transport of moisture from within and out of the sample at higher heating rates. These results show that drying profiles differ for fecal types and moisture can be removed from all fecal samples, provided there are no factors limiting drying kinetics. A relatively low heating rate favors drying above the lower critical moisture content but a higher heating rate would be required at relatively low moisture levels to reduce drying time. Further studies, using combined imaging techniques and computational methods, are required to ascertain the exact mechanisms by which moisture is transported through the different fecal solids. Evolved gas analysis can be conducted at low heat temperatures to ascertain that drying is due to loss of moisture and not for highly volatile compounds.

### 3.2. Effect of sample blending

**Figure 3.**
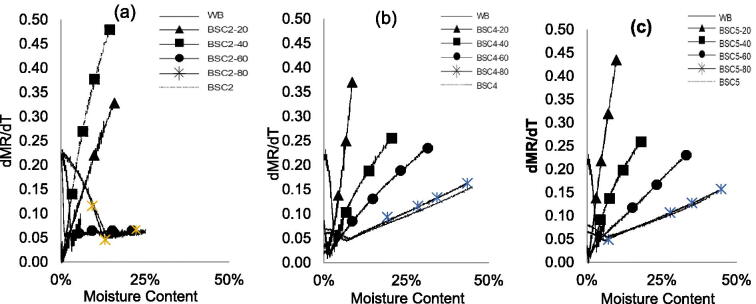
Non-isothermal drying curves for HF–WB blends and in comparison, with HF and WB at 10 K/min with respect to moisture ratio vs. moisture content: (a) BSC2, (b) BSC4, and (c) BSC5.

The moisture content of the samples decreased without drying by blending 20–80 wt.% of HF with WB. As shown in Table 1, the moisture content for BSC2-X blends decreased from 54% to 25% (BSC2-80), 21% (BSC2-60), 16% (BSC2-40), and 14% (BSC2-20), a corresponding decrease of 53%, 61%, 70%, and 74% in moisture content. For BSC4-X and 5-X blends, the moisture content also reduced; however, at lower values. For BSC4-X blends, the moisture content reduced by 30%, 44%, 63%, and 84% while a reduction of 27%, 47%, 71%, and 84% was observed for BSC5-X blends, respectively. Following non-isothermal drying at a heating rate of 10 K/min, Figures 3(a–c) illustrate the effect of blending on the non-isothermal drying of HF. The blends for the different feces were compared to 100% HF and WB at the same heating rate (10 K/min).

**Figure 4.**
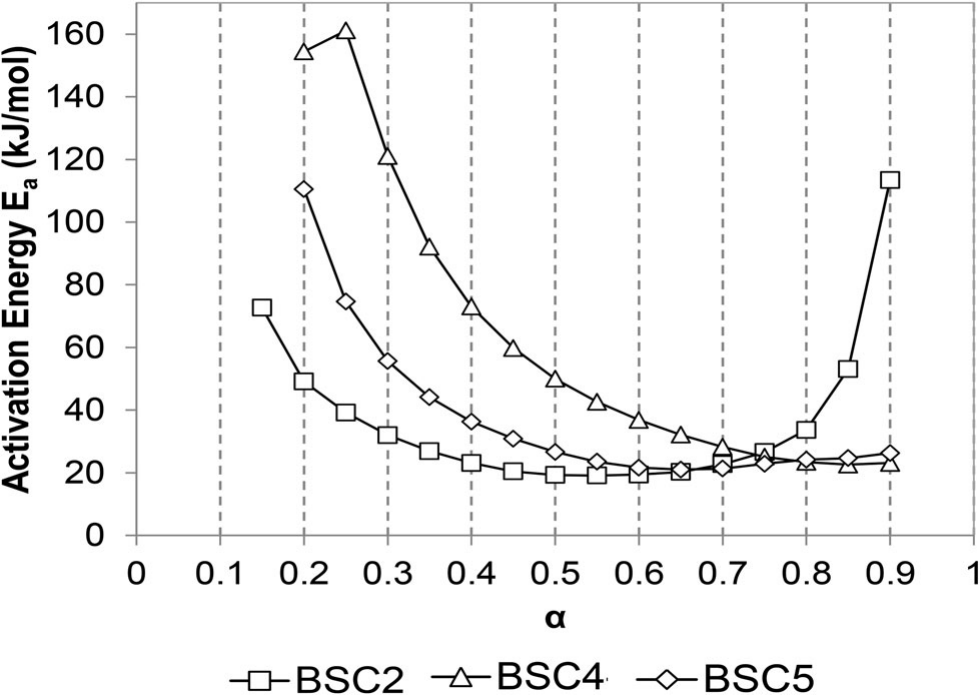
Activation energy, *E_a_*, distribution at different conversion degrees, *α* (OFW method).

Figure 3(a–c) confirm that blending HF with WB improves the drying characteristics of HF. The drying rates significantly improved, and the drying time reduced for all the blend types. The drying rate curves show that BSC2-80 blend had a similar drying curve to parent material while other blends (BSC2-20, -40, and -60) exhibited different drying characteristics. For BSC2-80, drying rate slightly decreased up to moisture levels of 18 wt.%, and then progressively improved until moisture was totally removed. For the BSC2-20, -40, and -60, drying rates were initially high (at about 0.30 mg/min) and gradually decreased over time. Similar trends are observed for BSC4-X and 5-X blends with BSC4-20 and BSC5-20 having the most improved dying rates (Figure 3(a,b)). A distinction in the moisture profile for the different blends cannot be inferred and outside of the scope of this work. It is, however, worth mentioning that the drying curves for WB100 and HF100 exhibit converse drying curves, but the drying curves for the blends are reflections of both materials. Seader et al.^[^^22^^]^ describe the phenomenon for the removal of moisture via capillary flow and diffusion. The capillary flow of moisture exhibits a concave characteristic: upward near the exposed surface and downward near the opposed surface with a point of inflection in between. The flow of moisture via diffusion is described as concave downward throughout, with other factors such as shrinkage, etc. playing a role on diffusivity and the amount of moisture removed. The point of inflection reflects the critical moisture content but is subject to change, depending on the ease of moisture transport and diffusion through the solid, and relates to material properties such as porosity, thickness, and the magnitude of mass transfer limitations.^[^^28^^]^

### 3.3. Kinetic analysis

Figure 4 shows the activation energy, *E_a_*, vs. the degree of conversion, *α*, for each HF type using OFW method (model-free). The activation energy was calculated through corresponding temperatures at a fixed degree of conversion and heating rate. Degrees of conversion ranging from 15% to 90% were considered in this work. Figure 4 reveals the multi-step kinetics of the drying process. In the case of BSC4 and BSC5, the variation of activation energy with the degree of conversion can be divided into two parts. The *E_a_* values decreased for *α* ≤ 0.7, and the values were higher in the case of BSC4. For *α* > 0.7, the energy of activation was steady and around 24 kJ/mol for both samples. The lower activation energies at higher degrees of conversion may be related to a reduction of bound water and increase in temperature of the sample.^[^^29^^]^ In agreement with observations in Section 3.2, BSC2 behaved differently. Thus, three parts can be identified in the variation of activation energy with the degree of conversion; *E_a_* was ∼73 kJ/mol at *α* = 0.15, decreased to a minimum value of ∼19 kJ/mol at *α* = 0.5, and then increased up to was ∼113 kJ/mol at *α* = 0.95. Comparing the three HF types, *E_a_* values followed the order of BSC4 > BSC5 > BSC2 for *α* ≤ 0.75, and BSC2 > BSC4 ≈ BSC5 for *α* > 0.75.

Table 2 summarizes the kinetic parameters obtained from CR method (model-fitting) of each range of conversion degrees considering each different heating rate and HF type. It can be observed that for all HF and heating rates the *E_a_* and *A* values decreased when the degree of conversion increased. Moreover, *E_a_* decreased when the heating rate increased at a given *α* value. This may be related to the fact that drying occurrence was shifted towards higher temperatures when the heating temperature increase leading to higher temperatures inside the sample. Comparing to the *E_a_* values obtained from OFW method and CR method, BSC2 and BSC5 were in good agreement. Nevertheless, in the case of BSC2, CR method at *α* > 0.70 did not reflect an increase in *E_a_* opposite to OFW method. For BSC4 samples, *E_a_* values obtained from OFW were higher than those obtained from CR method for any heating rate.

**Table 2.** Non-isothermal drying parameters of human feces by CR method.

Heating rate (K/min)	Sample	*α*	*g*(*α*)	*R*^2^	*E_a_* (kJ/mol)	*A* (K^2^/min)
10	BSC2	0.15–0.20	[1 − (1 − *α*)^1/3^]^2^	0.998	45.91	10^4.8^
		0.20–0.40		0.988	25.94	10^1.1^
		0.40–0.70	*α*^2^	0.940	21.55	10^1.4^
	BSC4	0.20–0.25	[1 − (1 − *α*)^1/3^]^2^	0.999	70.69	10^9.8^
		0.25–0.35		0.997	50.16	10^6.2^
		0.35–0.45		0.997	34.85	10^3.5^
		0.45–0.65		0.995	22.29	10^1.4^
		0.65–0.90		0.942	19.06	10^0.8^
	BSC5	0.20–0.25	[1 − (1 − *α*)^1/3^]^2^	0.999	44.57	10^4.8^
		0.25–0.45		0.991	25.94	10^1.6^
		0.45–0.75	*α*^2^	0.929	20.49	10^0.7^
5	BSC2	0.15–0.20	[–ln (1 − *α*)]^1/4^	0.698	72.10	10^11.7^
		0.20–0.40	[–ln (1 − *α*)]^1/2^	0.985	22.81	10^2.8^
		0.40–0.70	−ln (1 − *α*)	0.980	19.49	10^2.0^
		0.70–0.95	[1 − (1 − *α*)^1/3^]^2^	0.980	11.88	10^−0.4^
	BSC4	0.20–0.25	[1 − (1 − *α*)^1/3^]^2^	0.999	90.12	10^12.7^
		0.25–0.35		0.997	62.13	10^7.9^
		0.35–0.45		0.998	41.46	10^4.4^
		0.45–0.65		0.993	22.29	10^1.4^
		0.65–0.90		0.974	20.80	10^0.8^
	BSC5	0.20–0.25	*α*^3/2^	0.998	96.56	10^15.3^
		0.25–0.45	−ln (1 − *α*)	0.990	41.24	10^5.9^
		0.45–0.75	(1 − *α*) ln (1 − α) + α	0.966	24.78	10^2.4^
		0.75–0.90	[1 − (1 − *α*)^1/3^]^2^	0.988	9.77	10^−0.7^
1	BSC2	0.15–0.20	[−ln (1 − *α*)]^1/2^	0.998	57.71	10^8.3^
		0.20–0.40		0.987	30.21	10^3.4^
		0.40–0.70	1 − (1 − *α*)^1/2^	0.968	20.24	10^1.1^
		0.70–0.95	[1 − (1 − *α*)^1/3^]^2^	0.981	12.37	10^−0.9^
	BSC4	0.20–0.25	[−ln (1 − *α*)]^1/2^	0.999	144.03	10^23.7^
		0.25–0.35		0.997	99.14	10^15.9^
		0.35–0.45		0.998	66.20	10^10.1^
		0.45–0.65		0.993	39.28	10^5.3^
		0.65–0.90		0.964	14.69	10^−21.8^
	BSC5	0.20–0.25	*α*^1/3^	0.998	88.43	10^14.0^
		0.25–0.45	[−ln (1 − *α*)]^1/4^	0.988	41.07	10^5.6^
		0.45–0.75	[−ln (1 − *α*)]^1/3^	0.968	18.66	10^1.5^
		0.75–0.90	[1 − (1 − *α*)^1/3^]^2^	0.958	24.01	10^1.6^

At 10 K/min, the drying reaction mechanism, *g*(*α*), was the same for all type of HF considered and degree of conversion. On the contrary, at 1 and 5 K/min the best-fitting drying mechanism varied with the type of HF and across the degree of conversion, although certain resembles could be observed among samples and conversion degrees. This observation points that in units where the operation ability to respond to changes in the type of HF is limited, such as the NMT, it may be advantageous to carry out the drying step at relatively high heating rate.

## 4. Conclusions

In this work, an experimental investigation of the non-isothermal drying behavior of HF was carried out by means of TGAs. HF (Types 2, 4, and 5 (according to the BSC)) were used as feedstock to assess potential differences on their drying behavior. Considerations were given to various heating rates and blends of HF with wood biomass. The results show that drying profiles differ for fecal types and moisture can be removed at lower temperatures, provided there are no factors limiting drying kinetics. At 1 K/min and 323 K, the BSC4 and 5 fecal types lost ∼80 wt.% of the total moisture; but, the BSC2 still contained more than 50% of its initial moisture. The slow heating rate allowed effective heat transfer into the inner part of the sample and favored a progressive release of moisture but at the expense of drying time at moisture levels below 20 wt.%. At higher heating rates (10 K/min), the gradual release of moisture was limited, with a significant amount of delay in moisture removal, but drying rate improved below the lower critical moisture content. The different behavior among the samples was attributed to compact nature of solids and its influence on the migration of water. The drying reaction mechanism for BSC2 is likely different to BSC4 and 5. In all the sample types, drying did not involve a constant rate period prior to the dominant falling rate regime, which suggests that the initial moisture levels in the samples are below the “critical moisture content” and limited free water is present in the material that can be removed by dewatering. Blending with wood biomass improved the drying characteristics of HF. The results reported in this paper provide an introductory understanding of the drying pattern of HF. Further studies, using combined imaging techniques and computational methods, are required to ascertain the exact mechanisms by which moisture is transported through the fecal solids. The results are relevant for designing a drying unit operation for HF and can be applied to appropriately design non-sewered sanitary systems with in-situ thermal treatment.
